# Evaluation on treatment of sustained low-efficiency hemodialysis against patients with multiple organ dysfunction syndrome following wasp stings

**DOI:** 10.1186/s12882-019-1428-5

**Published:** 2019-07-03

**Authors:** Ting-Ting Ye, Rong Gou, Ya-Ni Mao, Jian-Ming Shen, Dong He, Yan-Yan Deng

**Affiliations:** Department of Nephrology, Renmin Hospital, Hubei University of Medicine, No. 39 Middle Chaoyang Road, Shiyan, 442000 Hubei China

**Keywords:** Wasp stings, Multiple organ dysfunction syndrome, Sustained low-efficiency hemodialysis

## Abstract

**Background:**

To evaluate the treatment of sustained low-efficiency hemodialysis (SLED) against patients with multiple organ dysfunction syndrome (MODS) following wasp stings.

**Methods:**

Clinical data of 35 patients with MODS following wasp stings were retrospectively analysed. These patients were divided into three groups according to the treatment strategy used: 1) hemodialysis (HD) group, 2) continuous veno-venous hemofiltration (CVVH)/HD group, and 3) SLED/HD group. The clinical parameters, treatment outcome, and safety findings were compared among the three groups.

**Results:**

The recovery rate (76.92% vs 77.78% vs 91.67%, *p* = 0.621) and mortality rate (15.38% vs 11.11% vs 8.33%, *p* = 0.999) were similar among the three groups. When compared to the HD group, patients treated with CVVH/HD or SLED/HD required a shorter period of time to enter into polyuria stage [(24.7 ± 4.3) days vs (20.2 ± 4.7) days vs (18.2 ± 3.0) days, F = 9.11, *p* = 0.0007], and required a shorter time for serum creatinine to return to normal [(45.7 ± 13.4) days vs (33.1 ± 9.4) days vs (31.9 ± 9.8), F = 5.83, *p* = 0.0069]; while such parameters had no significant differences between SLED/HD group and CVVH/HD group. The adverse events of hypotension and arrhythmia were found in the HD group, while no adverse events were reported in the SLED/HD and CVVH/HD groups. There was no significant difference in the cost of blood purification treatment between the SLED/HD group and HD group.

**Conclusion:**

The use of SLED, CVVH and HD provided a comparable recovery and survival rates in patients with MODS secondary to wasp stings. Compared to HD, the use of SLED is recommended as a treatment strategy because of the efficacy on recover of renal function, satisfactory safety outcome, as well as the reasonable treatment cost.

## Background

The incidences of wasp stings are frequently reported in rural areas. The outcomes can range from a mild local anaphylactic reaction to a severe systemic reaction, like multiple organ dysfunction syndrome (MODS) [1, 2]. The severe wasp sting injuries have been occurring more often in countries including India, Vietnam, Thailand, Malaysia, and China [2, 3]. The underlying mechanism of wasp sting induced reaction may comprise the direct toxic effect of venom and immune inflammatory reaction to the composition of venom. In China, most patients with multiple wasp stings presented with toxic reactions and MODS caused by the venom [3]. Currently, apart from hemodialysis, the most prevalent therapies used for patients with mass wasp attacks consist of the administration of antihistamines, corticosteroids, bronchodilators, vasodilators, bicarbonate, mannitol, adrenaline, and mechanical ventilation. However, most of these treatments seem to provide limited efficacy [4].

It is recognized that blood purification is an effective measure for removing toxic substances and inflammatory factors from the blood system. However, there are limited studies to evaluate the potential use of blood purification techniques for MODS secondary to wasp stings. Several techniques are available including hemodialysis (HD), continuous veno-venous hemofiltration **(**CVVH), and sustained low-efficiency hemodialysis (SLED). In most of the situations, the major indication for choosing CVVH over intermittent HD is hemodynamic instability. However, randomized trials have not shown the improvement of hemodynamic stability among patients treated with CVVH compared to HD [5]. In addition, the use of CVVH is more technically demanding and might be difficult to perform in rural areas where the medical facility is not optimal. SLED, on the other hand, is technically easier to perform and provides the same hemodynamic stability as CVVH, even in critically ill patients [6, 7]. In the current study we compare the use of HD and its combination with CVVH or SLED for treating patients with wasp sting induced MODS.

## Patients and methods

### Patient characteristics

Clinical data from 35 patients with MODS developed after wasp stings were retrospective reviewed. These patients were admitted to our hospital between 2004 and 2009. They were divided into three groups according to the treatment received: 1) HD group (*n* = 14); 2) CVVH/HD group (*n* = 9); and 3) SLED/HD group (*n* = 12).

### Treatments

The sting was removed and painkiller was given if severe pain was reported.

Blood purification therapy: **1)** HD group: hemodialysis was performed using 4008B hemodialysis machine with F60 filter (1.7m^2^, ultrafiltration coefficient of 40 ml/h x mmHg, inulin clearance of 98 ml/min, QB of 200 mL/min) (Fresenius Medical Care, Bad Homburg, German). After continuous treatment for 2 times, the treatment was switched to every other day. The duration of the HD dialysis was 4 h (Kt/V = 1.20 ± 0.09). Bicarbonate dialysis solution was used with flow rate of 500 mL/min. The blood flow was 200–250 ml/min. **2)** CVVH/HD group: multiFitrate Acute Therapy System was used (Fresenius Medical Care, Bad Homburg, German), and AV600S filter (1.4 m^2^, ultrafiltration coefficient of 49.8 ml/h x mmHg, inulin sieving coefficient of 1) was used for the dialysis. The blood flow was 150–180 ml/min; bicarbonate dialysis solution was administered pre-dilution (flow rate = 300 ml/h). The treatment was performed in 12–24 h/day (24 h, Kt/V = 2.25 ± 0.11); after 2 days of treatment, it was switched to every other day HD treatment. **3)** SLED/HD group: SLED treatment was performed with 4008S hemodialysis machine and AV600S filter (Fresenius Medical Care, Bad Homburg, German). Bicarbonate dialysis solution was used with flow rate of 300 mL/min. The blood flow was 150–180 mL/min, and the treatment time was 10 h (Kt/V = 1.4 ± 0.08). 10 mL intravenous supplementation of 10% calcium gluconate was given at the eighth hour of treatment to prevent hypocalcemia. The treatment was continued for 2 times and then switched to every other day treatment, with SLED alternating with HD. All patients from the three groups had the combined hemoperfusion-hemodialysis in the first two treatments. Blood purification treatment was ceased when polyuria was observed (urine volume > 2500 ml/d or > 1500 ml/d for those undergoing blood purification treatment).

### Measurement of clinical parameters

Clinical data from the three groups were compared; the data include the baseline characteristics, the time to enter polyuria stage, the duration for serum creatinine to return to normal, blood purification treatment-related adverse events, the clinical outcome, and the blood purification cost (excluding the cost of hemoperfusion and central venous catheterization). The following parameters were compared between Day 3 and Day 7: APACHE II (acute physiological and chronic health evaluation), white blood cell (WBC), haemoglobin (Hb), serum creatinine (Scr), serum urea (SU), total bilirubin (TB), alanine aminotransferase (ALT), creatine phosphokinase (CPK), creatine phosphokinase isoenzyme (CKMP). The APACHE II is a scoring system to measure the severity of disease in patients [8].

### Statistical analyses

The normally distributed measurement data were described as mean ± SD, and non-normally distributed data were presented as median and quartile range. One-way ANOVA or non-parametric rank-sum test were used to compare differences between three groups. If the difference was statistically significant, further comparison between two groups was performed using the Scheffe test or the Wilcoxon-Mann-Whitney test. Categorical data was analysed using Fisher’s exact test. Stata 8.0 software package was used for statistical analysis, and *p* < 0.05 was considered as statistically significant, two-tailed test was used.

## Results

### Comparison of baseline characteristics

We first compared the baseline demographic and clinical characteristics between the HD, CVVH/HD, and SLED/HD groups. There were no significant differences between the three groups in age, gender, and duration of disease onset to the first blood purification treatment. Other clinical parameters, including, APACHE II score, WBC, Hb, platelet (Plt), urine volume, Scr, Su, TB, ALT, LDH, CK, CKMP, PT, activated partial thromboplastin time (APTT), also had no significant differences between the three groups (Table 1).

**Table 1:** Comparisons of baseline characteristics among three groups

Parameters	HD group (*n* = 14)	CVVH/HD group (*n* = 9)	SLED/HD group (*n* = 12)	*P*-value
Age (year)	52.5(50–60)	55(52–59)	48.5(44–54.5)	0.169^*^
Gender (Male/Female)	8/6	5/4	5/7	0.759^*#*^
Duration of disease onset to blood purification (hr)	93.4 ± 13.9	96.8 ± 15.0	99.3 ± 17.2	0.622^△^
APACHE II score	20.29 ± 2.33	22.33 ± 2.06	21.67 ± 1.87	0.072^△^
Mean arterial pressure (mmHg) (1 mmHg = 0.133 KPa)	94.54 ± 27.17	88.48 ± 21.87	86.33 ± 21.38	0.669^△^
WBC (×10^9^/L)	28.41 ± 5.33	25.58 ± 4.81	27.19 ± 3.96	0.389^△^
Hb (g/L)	95.29 ± 19.44	90.11 ± 20.97	93.25 ± 18.79	0.827^△^
Plt (×109/L)	81.93 ± 16.53	83.80 ± 19.70	85.73 ± 17.37	0.773^△^
Urine volume (ml/h)	9.43 ± 3.64	8.18 ± 3.94	8.78 ± 3.21	0.725^△^
Scr (μmol/L)	779.2 ± 124.1	822.4 ± 140.9	804.8 ± 117.4	0.715^△^
TB (μmol/L)	62.42 ± 21.00	68.29 ± 18.04	66.09 ± 19.31	0.771^△^
ALT(U/L)	1320.9 ± 409.7	1296.0 ± 444.7	1127.4 ± 356.3	0.443^△^
LDH (U/L)	4992.8 ± 1422.9	5281.7 ± 1173.0	4771.7 ± 1322.0	0.688^△^
Creatine kinase (U/L)	21,506 ± 8977	19,889 ± 9032	18,346 ± 6828	0.631^△^
Creatine kinase-MB (U/L)	779.8 ± 228.8	737.9 ± 198.9	758.4 ± 207.8	0.899^△^
NT-proBNP (pg/mL)	3245.6 ± 1127.7	3196.2 ± 1205.3	3289.1 ± 1419.6	0.815^△^
PT(s)	24.69 ± 3.73	26.38 ± 4.49	23.06 ± 4.21	0.199^△^
APTT(s)	48.55 ± 12.89	46.56 ± 8.11	50.63 ± 9.21	0.681^△^

*Comparison between three groups,* *non-parametric rank sum test, #Fisher exact test,△One-way ANOVA

### Clinical outcome

In the HD group, two patients died during the study period, three patients fully recovered and were discharged, and nine patients were medically stable for discharge (7 patients recovered after follow-up, 1 patient had mild microscopic hematuria, and 1 patient had lost follow-up). In the CVVH/HD group, one patient died during the study period, 2 patients fully recovered and were discharged, and 6 patients were medically stable for discharge (5 patients were cured after follow-up and 1 patient had mild proteinuria). Lastly, in the SLED/HD group, 1 patient died, 4 patients fully recovered and were discharged, 7 patients were medically stable for discharged (all of them recovered after follow-up). There were no significant differences between the three groups in recovery rates (76.92% vs. 77.78% vs. 91.67%, *p* = 0.621) and mortality rate (15.38% vs. 11.11% vs. 8.33%, *p* = 0.999).

### Time for achieving polyuria and returning of serum creatinine to normal level

Next we compared the laboratory findings between the three groups. Significant differences in the time entering polyuria stage were found between three groups (24.7 ± 4.3 vs. 20.2 ± 4.7 vs. 18.2 ± 3.0 d, F = 9.11, *p* = 0.0007) using one-way ANOVA. Further comparisons were done between two groups, in which significant differences were found in HD vs. CVVH/HD group (*p* = 0.002), and HD vs. SLED/HD group *p* = 0.032). No significant differences were found between CVVH/HD vs SLED/HD group (*p* = 0.514).

To assess the time for the recovery of renal function, we measured the Scr levels. Significant differences between the three groups were found in the time for Scr to return to normal, in which patients in the HD group required the longest time (45.7 ± 13.4 vs. 33.1 ± 9.4 vs. 31.9 ± 9.8 d, F = 5.83, *p* = 0.0069). Further analyses showed significant differences between two groups: HD vs CVVH/HD (*p* = 0.046) and HD vs. SLED/HD (*p* = 0.015), while the differences between CVVH/HD and SLED/HD were not significant (*p* = 0.9722).

### Reported adverse events and cost related to blood purification

In the HD group, 4 patients (28.57%) had hypotension and 2 patients (14.29%) had arrhythmia, and such events were not observed in the CVVH/HD or SLED/HD groups. Significant differences among the three groups were found in the incidence of hypotension (*p* = 0.31) but not the arrhythmia (*p* = 0.324). Overall, both the CVVH/HD and SLED/HD treatments provided a satisfactory safety outcome.

The medical cost is an important factor that affects patient’s treatment choice. *The cost of blood purification discussed here included drugs, labor costs related to blood purification, consumables such as purification tube, filters, replacement liquid* etc. We found the cost of blood purification among the three groups was statistically significant (RMB$6798 ± 1323 vs. 15,995 ± 1424 vs. 7, 927 ± 1402; F = 139.15; *p* = 0.031), with the CVVH/HD treatment being the highest cost. When comparing between the two groups, significant differences were found in the HD vs. CVVH/HD groups (*p* = 0.0001) and SLED/HD vs. CVVH.HD groups (*p* = 0.0001), but not the HD vs. SLED/HD groups (*p* = 0.064).

### Differences of clinical parameters between groups after treatment

The comparisons of clinical parameters among three groups after treatment were summarized in Table 1. On Day 3 post-treatment, no significant differences were found among the three groups in Hb and ALT levels. APACHE II score, WBC, and Scr in CVVH/HD group and SLED/HD group were significantly lower than those in HD group (*p* < 0.05 or 0.01). There was no significant difference in TB, CK and CKMB between SLED/HD group and HD group. Also, no significant differences were found in APACHE II score, Hb, ALT, CK and CKMB among the three groups on Day 7 after treatment (*p* > 0.05). The WBC and TB between SLED/HD group and HD group were comparable, while Scr was significant lower than the HD group (*p* < 0.05) (Table 2). The data suggested that in the early stage of the disease, SLED can better improve the general condition and inflammatory response of patients when compared to HD. As the disease progress, the differences became minimal.

**Table 2:** Comparison of clinical characteristics among three groups after treatment

Parameters	Time	HD group (*n* = 14)	CVVH/HD group (*n* = 9)	SLED/HD group (*n* = 12)	*P*-value^*^
APACHE II score	Day 3	17.21 ± 2.12	14.44 ± 1.94^△^	14.92 ± 1.78^*#*^	0.0031
	Day 7	11.14 ± 2.35	9.22 ± 1.20	9.92 ± 1.73	0.0625
WBC(×10^9^/L)	Day 3	22.46 ± 2.61	18.53 ± 1.67^△^	19.60 ± 1.71^△^	0.0002
	Day 7	16.52 ± 4.32	12.15 ± 2.94^#^	13.83 ± 4.10	0.0384
Hb(g/L)	Day 3	79.64 ± 7.56	73.11 ± 6.75	76.17 ± 6.01	0.0935
	Day 7	68.64 ± 10.31	66.11 ± 6.83	65.33 ± 6.43	0.5759
Scr (μmol/L)	Day 3	517.6 ± 76.7	434.3 ± 49.3^#^	421.1 ± 50.0^△^	0.0007
	Day 7	358.3 ± 45.6	321.2 ± 35.9	313.0 ± 49.0^*#*^	0.0340
TB (μmol/L)	Day 3	50.88 ± 10.58	38.61 ± 4.68^△^	43.26 ± 7.52	0.0049
	Day 7	33.13 ± 8.36	21.68 ± 5.27^△^	27.93 ± 6.62	0.0027
ALT(U/L)	Day 3	930.6 ± 181.6	863.9 ± 189.7	830.0 ± 154.3	0.3159
	Day 7	599.4 ± 181.9	513.2 ± 190.5	561.5 ± 198.6	0.5727
CK(U/L)	Day 3	20,487 ± 7236	14,325 ± 3527^#^	18,659 ± 4193	0.0436
	Day 7	1553 ± 738	1162 ± 611	1234 ± 651	0.3302
CKMB(U/L)	Day 3	556.6 ± 75.7	477.5 ± 70.6^#^	531.4 ± 59.2	0.0384
	Day 7	194.6 ± 40.0	175.0 ± 38.5	168.4 ± 42.3	0.2211

One-way ANOVA was used to analyse the differences among the three groups; the Scheffe method was used to test the difference between the two groups. Compared with HD group, * < 0.05 and ^△^ < 0.01

## Discussion

The incidences of wasp stings have occurred worldwide, especially in developing countries [9, 10]. In contrast to the occasional incidence in previous reports, the wasp stings have been occurring more frequently in China, causing a considerable mortality among victims [3, 11]. Patients with wasp stings reported in developed countries were usually suffered from a single sting, and an anaphylactic reaction is the main clinical feature of such patients. In China, however, patients were attacked by a crowd of wasps, resulting in severe hemolysis and rhabdomyolysis that greatly injured the kidneys [3]. Blood purification therapy is thus often applied to these patients. The current study showed that the three treatment strategies (HD, CVVH/HD, and SLED/HD) could provide a similar mortality and recovery rates. A previous case report described the successful outcomes after using CVVH to treat MODS secondary to wasp attacks [12]; nevertheless, a meta-analysis study did not find the survival advantage of using CVVH over HD to treat MODS patients with acute renal failure [13]. Our study also suggested that the use of SLED, CVVH, or HD provided similar clinical outcomes when treating patients with wasp sting induced MODS.

Renal failure is frequently observed in patients with wasp sting. These patients usually presented acute tubular necrosis or acute interstitial nephritis [14], and oliguria is rapidly observed with electrolyte imbalance. Blood purification treatment is thus important of these patients. Among the different blood purification methods, CVVH provided several advantages, including the improved hemodynamic stability that will allow better clearance of small−/macro-molecule of metabolites and recovery of renal function. On the other hand, HD has advantages of flexibility, cost-effectiveness, and is effective in removing small molecules such as potassium. It is also beneficial for reducing bleeding caused by anticoagulation. For critically ill patients, there has been no consensus on the ideal treatment that should be used, but combination treatment strategy has been suggested [15]. SLED provides advantages of both CVVH and HD. It is administered using conventional dialysis technology but over a prolonged period, thereby allowing for gradual removal of fluid with less hemodynamic perturbation [16–18]. For patients with critical acute kidney injury requiring renal replacement, the use of SLED and CVVH provides similar outcome in terms of overall mortality and renal function recovery [18].

The safety profiles of patients treated with SLED/HD or CVVH/HD were similar, in which there were no adverse events reported during the blood purification process. In addition, no significant difference was found between the two groups entering polyuria stage and the time when Scr returned to normal, indicating both of the blood purification methods could promote the recovery of renal function at about the same time. In comparison between SLED/HD group and HD group, patients in the SLED/HD group required a shorter time period to enter into the polyuria stage and had the Scr returned to normal level, also the incidence of hypotension was reduced. This could be due to the prolonged treatment time. Indeed, a study showed that the prolonged HD treatment could increase the clearance of urea and provide a more stable hemodynamic, when compare to the conventional HD [19]. Overall, the use of SLED/HD or CVVH/HD could provide a similar efficacy in renal function recovery.

The release of large amount of inflammatory mediators may contribute to the development of MODS [3, 20]. We found that on Day 3 of the treatment, patients in the SLED/HD group had a lower APACHE II score and a lower level of WBC when compared to the HD group, suggesting patients treated with SLED had a better improvement in general health condition and inflammatory reactions.

The cost of the treatment is an important factor when considering a treatment, especially for patients in rural area. It is well known that the expense of SLED treatment is higher than that of the HD. However, we found that in the current study the expenses are comparable between SLED/HD group and HD group. Both of these treatments had a lower cost than the CVVH/HD. The patients treated with SLED/HD required a shorter time period to enter into the polyuria stage and had a reduced number of treatments when compared to that of the HD group. Both of these factors contributed to the reduced cost of SLED treatment.

## Conclusion

The current study had small sample size and was retrospective in nature. A further study with a randomized control group is needed to validate the results. In summary, the use of SLED, CVVH or HD in treating MODS secondary to wasp stings provided a similar survival and recovery outcome. However, when compared to HD treatment, the use of SLED could provide a better recovery of renal function and improvement of general health condition at a reasonable cost. As such, we recommend the use of SLED as a treatment strategy for patients with MODS induced by wasp stings.

## Data Availability

The datasets generated and analyzed during the current study are available from the corresponding author on reasonable request.

## Abbreviations

ALT: Alanine aminotransferase
CPK: Creatine phosphokinase
CVVH: Continuous veno-venous hemofiltration
Hb: Haemoglobin
HD: Hemodialysis
MODS: Multiple organ dysfunction syndrome
Scr: Serum creatinine
SLED: Sustained low-efficiency hemodialysis
SU: Serum urea
TB: Total bilirubin
WBC: White blood cell
